# Role of Pure Technetium Chemistry: Are There Still Links to Applications in Imaging?

**DOI:** 10.1021/acs.inorgchem.3c01620

**Published:** 2023-07-07

**Authors:** Roger Alberto

**Affiliations:** Department of Chemistry, University of Zurich, Winterthurerstrasse 190, CH-8057 Zurich, Switzerland

## Abstract

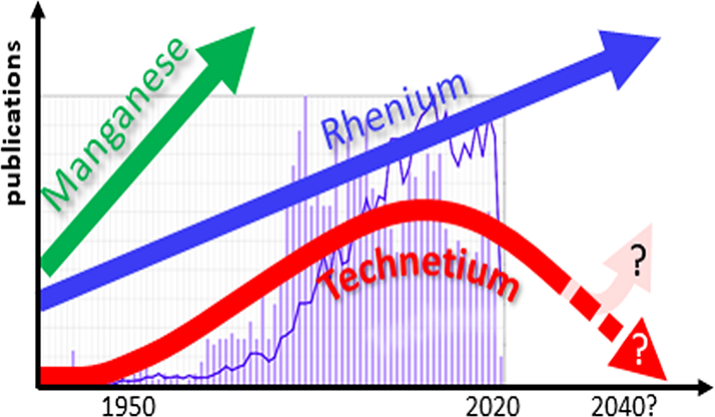

The discovery and development of new ^99m^Tc-based radiopharmaceuticals or labeled drugs in general is based on innovative, pure chemistry and subsequent, application-targeted research. This was the case for all currently clinically applied imaging agents. Most of them were market-introduced some 20 years ago, and the few more recent ones are based on even older chemistry, albeit technetium chemistry has made substantial progress over the last 20 years. This progress though is not mirrored by new molecular imaging agents and is even accompanied by a steady decrease in the number of groups active in pure and applied technetium chemistry, a contrast to the trends in most other fields in which d-elements play a central role. The decrease in research with technetium has been partly counterbalanced by a strong increase of research activities with homologous, cold rhenium compounds for therapy, disclosing in the future eventually a quite unique opportunity for theranostics. This Viewpoint analyzes the pathways that led to radiopharmaceuticals in the past and their underlying fundamental contributions. It attempts to tackle the question of why new chemistry still does not lead to new imaging agents, i.e., the question of whether pure technetium chemistry is still needed at all.

## Abstract 2

The declining or at least stagnating activities in pure technetium chemistry impact the development of ^99m^Tc-based new imaging agents and thus its relevance for nuclear medicine. This Viewpoint analyzes past tracks to successful radiopharmaceuticals, the current stage of research, possible reasons for the decline, and future opportunities from the parallel rise of rhenium chemistry.

## Introduction

**Figure 1:**
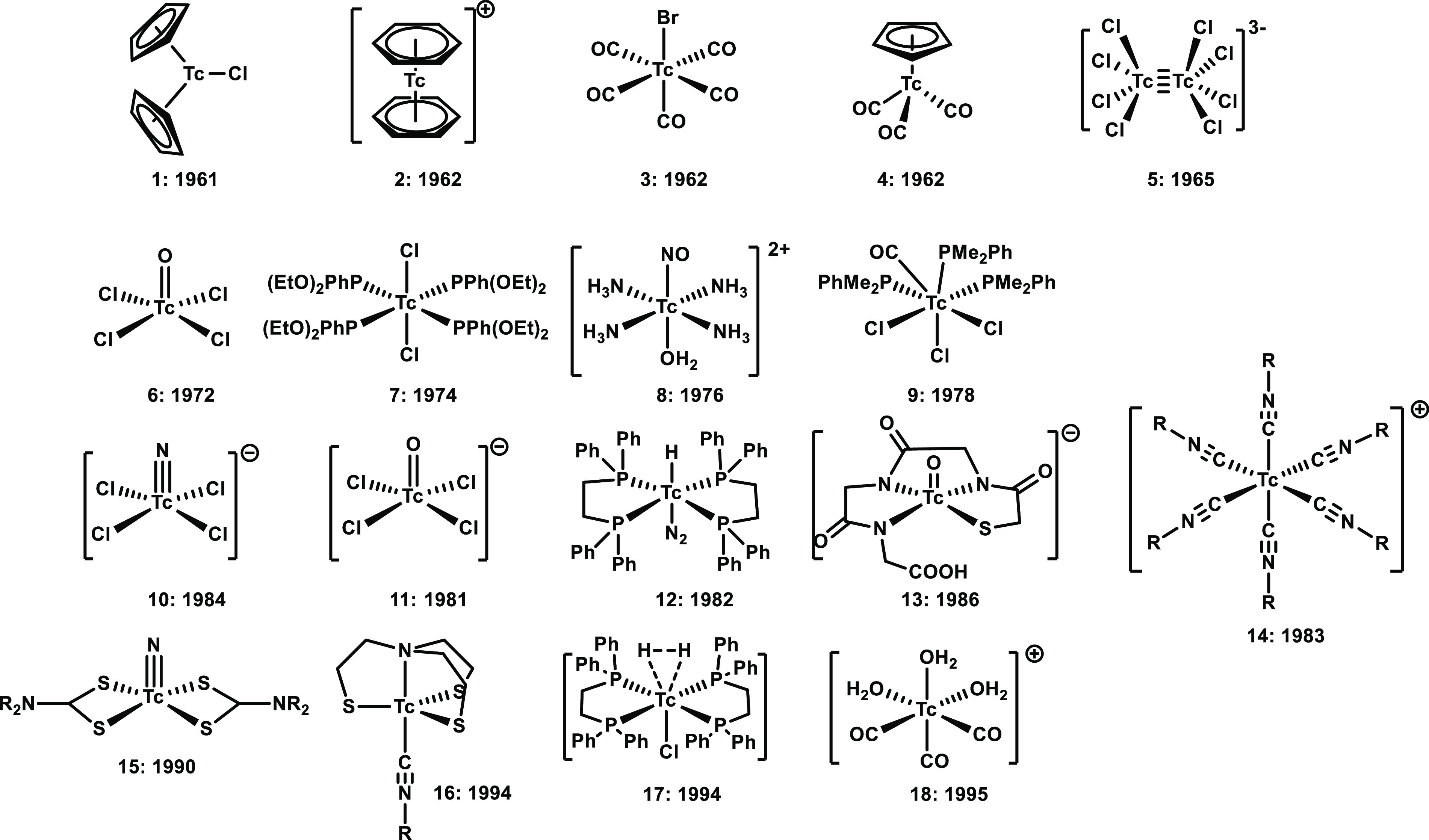
Fundamental compound (classes) and complexes reported in the 20th century: 1–4,^18−21^ 5,^17^ 6,^22^ 7,^23^ 8,^14^ and 9–18.^24−33^

The discovery and market-introduction of a commercial ^99^Mo/^99m^Tc generator together with the availability of macroscopic amounts of ^99^Tc have sparked enthusiastic progress in fundamental investigations about the chemistry of this element, essentially since the early 1960s.^1−6^ The close-to-ideal decay properties for imaging purposes such as its relatively short-lived half-life time of 6 h and the low price of ^99m^Tc for nuclear medical imaging purposes were and are a driving force for an in-depth understanding of the chemistry of technetium.^7^ However, the 6 h half-life time is too short and the solutions are too dilute for allowing unambiguous characterization of compounds eventually prepared with it. Fortunately for chemistry, nuclear fission in reactors produces the ground-state isomer ^99^Tc in large, macroscopic amounts.^8^ Because ^99^Tc has a half-life time of about 212000 years, chemistry can practically be done at the gram scale. Under strict consideration of radiation safety rules, compounds can be fully characterized with X-ray structure analysis, NMR, elemental analysis, and other common analytical methods. Besides fundamental insights, the high-performance liquid chromatography (HPLC) retention time’s comparison with what is obtained then with ^99m^Tc assesses the authenticity of the metastable compound. In this advent period starting in the 1960s, numerous fundamental technetium complexes, coordination and organometallic compounds, were synthesized and fully characterized. To select a few, the binary and ternary halide complexes [TcF_6_]^9^ and [Cat]_2_[Tc^IV^X_6_]^10^ as well as other classical starting compounds such as [Cat][Tc^V^OX_4_],^11^ [Cat][Tc^VI^NX_4_],^12,13^ [Tc(NO)(NH_3_)_4_(OH_2_)]^2+^,^14^ [Tc^0^_2_(CO)_10_],^15^ [Cat]_2_[Tc^III^_2_X_8_]^16,17^ and others were among the first to be prepared. In parallel, radiopharmaceutical chemistry did flourish based on some of these building blocks via the subsequent preparation of water- and air-stable coordination compounds with ^99^Tc and partly with ^99m^Tc (Figure 1).

Still, many compound classes were not studied, and it was not until the 21st century that, e.g., binary halides of the middle and lower oxidations states were prepared and comprehensively characterized (*vide supra*).^34−37^ They are essential for completing knowledge of the manganese triad and for systematizing vertical, horizontal, and diagonal trends but even more for acting as starting materials for extended, synthetic coordination and organometallic chemistry.

### Chemistry and Radiopharmaceuticals

**Figure 2:**
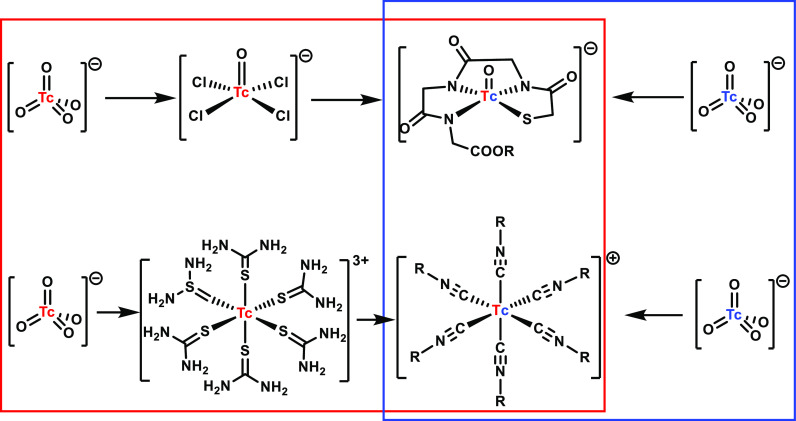
Interlink of pure chemistry (red, ^99^Tc) and application (blue ^99m^Tc). The preparation under any conditions is followed by a synthesis from [^99m^TcO_4_]^−^ in one step and in saline.

Of particular interest is the search for and the preparation and investigation of water-stable complexes because they have potential in radiopharmaceutical applications as *de novo* compounds (first-generation radiopharmaceuticals) or as labels attached to targeting biomolecules or pharmaceuticals (second generation). To reach an applicable radiopharmaceutical, a distinct interplay between pure and applied chemistry was and is indispensable, starting from preparing the compound under any conditions and converting its preparation into a kit formulation. Accordingly, pure research was plentiful in the 1980s and 1990s and was clearly, but not only, inspired and followed by a distinct interest in applications coming from the life sciences and the growing importance of ^99m^Tc. Along this thrust, chemistry especially around the [Tc^V^=O]^3+^, [O=Tc^V^=O]^+^, and [Tc^V^≡N]^2+^ cores was investigated in all their facets. As a follow-up, different medicinally important imaging agents were developed and market-introduced, e.g., TcMAG3 (Figure 2), Tc-HMPAO, and Tc-ECD.^38−40^ These agents are still in use under the trade names Technescan, Ceretec, and Neurolite. Their successes were based on detailed chemical studies and boosted by the enthusiasm from companies interested in ^99m^Tc radiopharmaceuticals. Following a hypothesis that cations would be transported by the Na^+^/K^+^ATPase pump in the myocardium, it was shown during the exploration of lower-valent technetium compounds that monocationic complexes of Tc^III^ and the general formula [TcCl_2_(P^2^)_2_]^+^ with P^2^ = bidentate phosphanes showed excellent heart uptake and persistence properties in animals.^41−47^

This, in turn, initiated a vivid development of chemistry in the lower oxidation states, paralleled by all sorts of basic studies such as, e.g., extensive electrochemistry.^48−50^ Discovered and developed from pure fundamental interests, these Tc^III^ complexes were then turned into an applicable synthesis with ^99m^Tc. Despite showing excellent *in vivo* behavior in animals, the Tc^III^ complexes were finally found to be reduced in humans to neutral Tc^II^ species, which did not accumulate in the myocardium as expected (since neutral) and thus did not fulfill the conditions for *in vivo* application.^47^ This failure did not have a negative influence on pure research, and numerous publications appeared with extensive results about the widespread chemistry of Tc^III^ and lower oxidation states.^24,51−56^ Shortly after, Davison et al. published their hexaisonitrile complexes [Tc(CN-R)_6_]^+^, first as a fundamental class of organometallic compounds. Soon after, inspired by the cation hypothesis, they reported about the conversion of ^99^Tc into an applicable aqueous synthesis with ^99m^Tc (Figure 2).^29,57−59^ With R = −CH_2_(CH_3_)_2_OMe, this cation became the most successful radiopharmaceutical, market-introduced under the trade name Cardiolite. Cardiolite remains probably the most important ^99m^Tc-based radiopharmaceutical, although its patent ran out many years ago. Novel chemistry preceded commercial success, and the final formulation from applied chemistry to prepare it from a saline generator elute was of the highest innovation. The ^99^Tc complex was originally prepared along classical methods, and it was not clear whether a preparation in saline would work.^29,60^

Cardiolite is an extremely useful radiopharmaceutical that has saved many lives. It is studied not only in the context of myocardial imaging but also in multiple drug-resistance studies and breast cancer.^61−63^ Despite these advantages, it is hardly applicable for the labeling of targeting molecules (second generation) because the isonitrile ligands are so robustly bound that they are not easily replaced under applicable conditions. Because radiopharmacy focused on *de novo* molecules at that time, this was not the primary intention anyway. The opportunities emerging from pure chemistry interests were obviously in agreement with this concept. The labeling of biological (macro)molecules such as antibodies came into focus during the late 1980s and 1990s.^64−70^ Abrams and co-workers introduced the so-called “hynic approach”, which allowed a convenient and straightforward labeling of biological molecules such as proteins and peptides, an approach that is still followed to date.^71−79^ Despite being a convenient methodology, even after many years, it remains unclear what the label looks like because it needs various coligands to stabilize the core. Model complexes with ^99^Tc or rhenium, matching the ^99m^Tc behavior, do not exist, and the approach does not work with ^188^Re for radiotherapy. The most likely structure is a diazenido linkage, but this has not been assessed because the preparation of analogous ^99^Tc or rhenium complexes for characterization was not yet successful, albeit a variety of rhenium and technetium complexes with metal–nitrogen multiple bonds were fully characterized, e.g., by Dilworth and co-workers.^80,81^

The 1970s, 1980s, and 1990s were thus a period of extensive and exciting technetium chemistry, including the clinical introduction of the most successful radiopharmaceuticals, and many of them are still in clinical routine. Dozens of research groups were active worldwide during these periods, and numerous new compounds with no immediate relevance for radiopharmacy were reported in parallel with extensive efforts of making their syntheses possible with ^99m^Tc. A pure understanding of their chemistries was the driving force, sometimes inspiring application but also vice versa; i.e., questions from applications led to innovations in pure and then applied chemistry. The knowledge gap between manganese and rhenium started to get filled up.

### Rhenium and Technetium

We note that many early inputs came indeed from rhenium chemistry, raising the question about the existence of homologous technetium compounds and their eventual physicochemical differences. Rhenium and technetium chemistries are often treated in parallel. The apparent similarities between the two elements, based on the textbook notion about 4d and 5d elements, coined the expression “matched pair” because most, but not all, of the complexes available for rhenium are also accessible with technetium, at least in the low but generally less so in the middle and higher oxidation states.^6^ This matched-pair concept has experienced a revival because the discovery of complexes based on the *fac*-[Re(CO)_3_]^+^ core are highly active against cancer cells.^82−84^ Because their ^99m^Tc homologues are easily prepared, the option of a theranostic application comes into reach and helps to eventually resurrect technetium chemistry.

**Figure 3:**
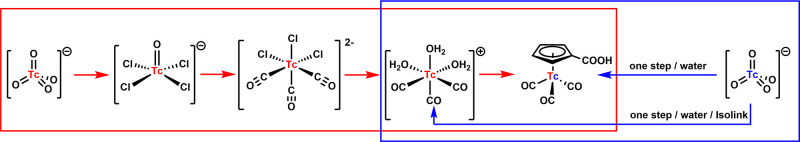
Access to a ^99^Tc piano-stool complex at 1 atm of CO (red)^33^ and the aqueous one-step synthesis for ^99m^Tc (blue).^99^

When the concept of *de novo* radiopharmaceuticals was extended to the second generation, substitutionally labile “building blocks” came more and more into focus. The concept of having fragments that can bind to multiple bifunctional ligands is attractive for the discovery and development of targeting radiopharmaceuticals by varying the coligands.^85−87^ Whereas the [Tc=O]^3+^ and [Tc≡N]^2+^ cores fulfill these conditions to a certain extent, their stabilization relies on multidentate chelators. In the late 1990s, the [Tc(OH_2_)_3_(CO)_3_]^+^ precursor was introduced, not particularly for radiopharmacy but rather from the desire to prepare carbonyl complexes at 1 atm of CO to study their basic chemistries (red track in Figure 3). It soon turned out that this water-stable and well-defined fragment would easily exchange the H_2_O ligands and bind to a plethora of a- and b-type ligands under the formation of highly inert complexes.^88^ Following the blue arrow in Figure 3, [^99m^Tc(OH_2_)_3_(CO)_3_]^+^ was provided in a kit formulation by Mallinckrodt as Isolink because it binds essentially to anything.^89,90^ Despite its convenience, only one complex made it ultimately through clinical phases^91,92^ for reasons not to be discussed here.^93^ Still, the preparation of the complex with ^99^Tc under water-free conditions inspired attempts to make it with ^99m^Tc from water, conditions under which the reaction with ^99^Tc exclusively gives ^99^TcO_2_ but no carbonyl complexes. This apparent difference between ^99^Tc and ^99m^Tc chemistry gave a fundamental perception, namely, that complexes with ^99^Tc not accessible in water may well be doable with ^99m^Tc, provided they are stable under these conditions. It also implies that chemistries between the two isomers are different (*vide infra*). “Attractive complexes, seemingly impossible to obtain from boiling water, are not out of reach for application purposes” is thus the core message for still pursuing pure technetium chemistry. As for the aforementioned cores, the availability of the *fac*-[^99^Tc(CO)_3_]^+^ fragment and its ^99m^Tc analogue entailed a plethora of new basic insights and opportunities, leading to such “nonaqueous” compounds as “agostic” hydrides, clusters, higher carbonyls, and many other classes in the first and second decades of the 21st century.^94−98^

### Current Situation

Having had publication activities climax in the 1990s and the early 21st century, contributions to basic synthetic technetium chemistry started to decline, despite the motivations and opportunities arising from, e.g., the carbonyl chemistry. When the stability and flexibility of the homologous *fac*-[Re(CO)_3_]^+^ core were realized, technetium chemistry began to inspire rhenium bioorganometallic chemistry.^100−103^ Until then, rhenium was not really considered as an element useful for the “metals in medicine” field, which focused essentially on the PGMs and gold or silver.^104^ Ever since, bioorganometallic chemistry of rhenium has had a steep rise, going beyond the application of ^188^Re for therapeutic purposes.^103^ This was probably for the first time when technetium inspired rhenium and not the other way around, as it used to be in the past. In the recent decade, rhenium has gained momentum and thus its place as a useful element for, e.g., classical cancer therapy, accompanied eventually by radionuclide therapy through its ^188^Re isotope.^82−84,105^ Along this line, the matched-pair concept experienced a revival because not only does rhenium serve as a surrogate of technetium in the lower oxidation states, but homologous complexes of the two elements might be combined for theranostics, i.e., the ^99m^Tc complex for imaging complemented by the cold rhenium complex for therapy or with ^188^Re for radionuclide therapy.^106^

Over the last 2 decades, the increasing dominance of positron emission tomography (PET) as a major tool of nuclear medicine affected the thrust for doing pure technetium chemistry. The improvements of cameras, the availability of PET nuclides beyond ^11^C and ^18^F, and the commercially available ^68^Ge/^68^Ga generator had an impact and made ^68^Ga, in particular, readily available.^107−111^ The chemistry with Ga^3+^ and other isotopes of the “3+ family” is admittedly much simpler because they do not involve any electron-transfer reactions. In parallel, industrial interest focused more and more on PET, which, in a broader sense, also allows for theranostics with, e.g., ^177^Lu as a therapeutic radionuclide, complementing ^68^Ga or ^111^In as an imaging tool.^112−115^ Consequently, the incentive for technetium chemistry decreased, and essentially no new building blocks or simple *de novo* complexes with the potential for radiopharmacy emerged, with a few exceptions.^116−118^ Whereas labeling of biomolecules or simple drugs with any of the above-mentioned precursors is a rich field, only a handful of groups are left worldwide dedicated to pure chemistry or ultimately for molecular imaging purposes since the 2010s. The number of publications in the classical platform for this chemistry, *Inorganic Chemistry* of the ACS, has shrunk since the early 2000s or at least stagnated. We note from a “Web of Science” analysis that biological *in vitro* or *in vivo* studies of ^99m^Tc imaging agents did not decline in contrast, essentially all applying the old concepts. With a few exceptions, Tilmanocept being probably the most prominent exception,^119,120^ no new imaging agents have been commercialized or are in phased clinical trials according to clinicaltrials.gov. A substantial part of basic technetium chemistry is nowadays focused on environmental aspects and nuclear waste treatment, where it has, e.g., been shown that the [^99^Tc(CO)_3_]^+^ core might play an important role^121,122^ but also the properties of HTcO_4_ have finally been established.^123^ Organometallic chemistry has been developed to the very low oxidations states by Abram et al., extending the knowledge from and comparing it with that of neighboring elements.^124−126^

### Newer Developments

**Figure 4:**
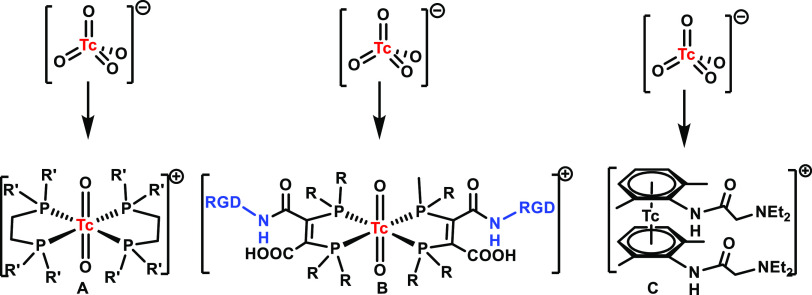
Three labeling approaches: *de novo* compounds (A) shown with the compound Myoview,^130^ the application of this platform to targeting peptides (B; RGD),^131^ and the integrated approach shown with lidocaine (C).^132^

Following the incentive that pure chemistry is essential for new labeling concepts or building blocks, we recently introduced chemistry based on arenes as ligands. Complexes of the types [^99^Tc(η^6^-arene)_2_]^+^ or their rhenium homologues, standard organometallic compounds for many other d-elements, became surprisingly easily accessible for ^99m^Tc. Such sandwich complexes of rhenium and technetium with benzene were prepared at the advent of organometallic chemistry by Fischer and co-workers, but their subsequent chemistry was essentially neglected over the following decades.^19,127^ Attracted by the beauty and intrigued by a side note of Fischer’s seminal publication about water washing of the product, we revived the synthesis by following an optimized rhenium procedure.^128,129^ Initially of purely basic interest for complementing arene-organometallic chemistry of the two elements, the extreme stabilities of these complexes at any pH and temperature and under air immediately implied potential applications in radiopharmacy. As was often the case in the past and the message of this Viewpoint, a chemistry of fundamental origin revealed a potentially new concept for molecular imaging, namely, the direct coordination of Tc to the π system of phenyls (Figure 4).

**Figure 5:**
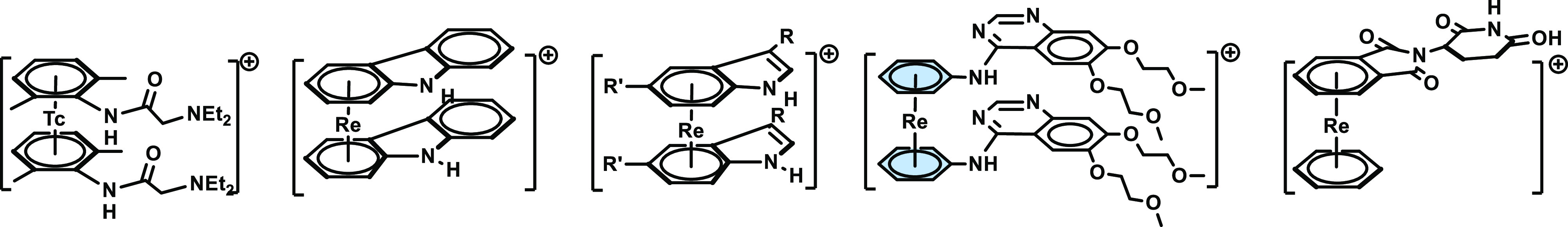
Examples of sandwich complexes with highly functionalized arenes available for ^99m^Tc and rhenium.

The syntheses of [^99^Tc(η^6^-arene)_2_]^+^, with the arene being benzene or simple alkylated arenes such as mesitylene, follows the classical Fischer–Hafner approach, useless for ^99m^Tc or for functionalized arenes. In the early 1990s, Wester and co-workers had already prepared these sandwiches with ^99m^Tc, according to the Fischer–Hafner procedure along a multistep route, out of the question for a routine application, but they made it.^133^ The motivation behind making them was the run for cationic *de novo* complexes for myocardial imaging (*vide supra*). They did not fulfill the expectations and were abandoned subsequently, albeit they were found to be apparently stable in biological systems. A more common synthesis applicable from water and with functionalized phenyls would probably have changed their relevance already at that time. Stability in water and air is uncommon for such sandwiches compared with the neighboring analogues: [Cr(η^6^-C_6_H_6_)_2_], which is air sensitive,^134^ and with one ring in [Ru(η^6^-C_6_H_6_)_2_]^2+^ being very labile.^133^ Going beyond benzene and polyalkylated arenes, we hypothesized that phenyl groups, ubiquitous in pharmaceutical lead structures, could directly be labeled through their π systems without needing bifunctional chelators attached to it. When [^99m^TcO_4_]^−^ was heated in saline, in the presence of an organic molecule comprising a substituted phenyl and ligand-dependent reducing agents, typically only one compound was obtained in variable yields. The solubility of the organic molecule is a decisive restriction.^135^ Water-soluble molecules such as paracetamol worked particularly well, in contrast to those with slight water solubilities. One HPLC peak does not mean much if the fully characterized rhenium or ^99^Tc analogue is not available for comparison. This was not the case for many of the more complex sandwiches. Thus, rarely found in the field of ^99m^Tc chemistry, well-defined ^99m^Tc complexes were accessible, but a synthetic approach to their analogues and homologues was unknown. A question from application thus entailed a fundamental challenge, namely, the preparation of highly functionalized rhenium or ^99^Tc bis(arene) complexes. We finally found that naphthalene in [Re(η^6^-C_10_H_8_)_2_]^+^ is replaced in low-to-good yields by phenyl-containing pharmaceuticals to yield [Re(η^6^-pharma)_2_]^+^ for comparison with the corresponding ^99m^Tc HPLC traces and confirmation of their structures.^132^ A few examples are shown in Figure 5. Whether such sandwich complexes will ever play a role in molecular imaging needs to be proven, but they open at least a new vista on ^99^Tc chemistry and complexes to be applied in molecular imaging following a different concept than what was previously known. This chemistry also represents one of the rare cases in which technetium inspired rhenium chemistry. The chemistry entailed a realm of further reactivities, leading to so-far-unknown fully solvated Re^I^, Re^II^, and Re^III^ complexes as well as piano-stool compounds of the [Re(η^6^-C_6_H_6_)(sol)_3_]^+^ type, showing promising reactivity patterns eventually useful in catalysis or for other purposes.^136−138^

### Kinetics versus Thermodynamics

It is evident that the same procedures in water but with macroscopic amounts of [ReO_4_]^−^ or [^99^TcO_4_]^−^ gave 0% yields in a sandwich complex, whereas with [^99m^TcO_4_]^−^, water, and soluble arenes, a single product with yields up to 90% was obtained. Why is this so? It is clear that, at nanomolar ^99m^Tc concentrations, kinetics may become the dominant factor, being much more product-deterministic than thermodynamics. Thermodynamics predicts that the TcO_2_·*x*H_2_O sink will be the end product, which was indeed found in all of the approaches described above. TcO_2_·*x*H_2_O is an oligo/polymer with rutile structure, predicted but never proven to exist with ^99m^Tc. At its nanomolar concentrations, the formation of higher, bulk polymers is kinetically unfavorable, although its formation is a standard explanation for “side products” immobile on thin-layer chromatography found in labeling experiments. Kinetic inaccessibility is the chance for accessing highly unlikely complexes such as those described above and is the chance and motivation of aiming at uncommon but still existing compounds under high dilution conditions. Kinetics is thus also the reason for the seemingly different chemistries of ^99m^Tc and ^99^Tc. Provided that stability under biological conditions or eventually even well-defined reactivities are given, the laws of kinetics may allow for the preparation and study of highly uncommon compounds, enabling unexpected fields of application.

### Future Technetium Chemistry

Technetium stands right in the middle of the transition elements. For all of its neighbors, catalytic processes have been described, useful for application or not. This field is void for technetium. Admittedly of pure academic interest, knowledge about catalytic activities could inspire processes for its neighbors, as is common in this field. More realistically, molecular imaging with ^99m^Tc is growing as the main application field. Still, the beauty and thrill of pure technetium chemistry over the decades, punctually exemplified above, seems not to be an incentive anymore. It is the viewpoint of this author that this situation must change to keep technetium’s relevance as a main contributor to nuclear medicine imaging alive. Some of the reasons for the decline, together with suggestions about how to change them, are as follows: (i) loss of economic interest of the pharmaceutical industry in technetium; (ii) lack of infrastructure; (iii) perspectives of doing ^99^Tc chemistry; (iv) nothing new to discover/develop.

The close connections between companies active in SPECT imaging and researchers were a strong driver for innovative new approaches. A good example was the search for cationic complexes for myocardial imaging, leading ultimately to Cardiolite and combining pure chemistry with application in molecular imaging. The support of DuPont at that time was essential for both. Since then, key companies have aligned their interest with PET nuclides or stopped their ^99m^Tc activities. This has entailed a growing innovation gap between academia and the private sector. Whereas the former turned to the labeling and biology of targeting molecules of direct interest for companies as mirrored by the growing number of such studies, the latter were no longer ready to support risky but eventually innovative technetium chemistry research projects, as was the case, e.g., with carbonyls and Mallinckrodt at that time. This led to a loss of attraction unless a research group had the luxury of not just relying on public or private funds. SPECT cameras have gotten better and better, and this may lead to a resumption of economic interest.

Working with radioactive elements such as technetium requires specially equipped laboratories and safety measures. However, ^99^Tc is a weak β emitter with a very long half-life time. The lowest level of laboratory equipment, according to European regulations, allows the convenience of working with ^99^Tc on the macroscopic level. Many laboratories have such equipment, e.g., for other radionuclides, which could be adapted to ^99^Tc.

The chemistry of technetium is a “niche field” due to its radioactive nature. Whereas for most of the other d-elements, multiple fields of applications are in reach; e.g., catalysis, (nano)materials, energy or life sciences, imaging, or waste treatment are small albeit important fields for justifying fundamental ^99^Tc chemistry, as outlined above. Given the competition from other radionuclides with apparently more convenient (facile) chemistry or superior biological behavior, the relevance of ^99^Tc chemistry shrinks accordingly. In combination with the first point, the perspectives for doing pure technetium chemistry for radiopharmacy are thus limited from several aspects, and it is not very attractive for young researchers to step into the field.

## Conclusion

It is the author’s concern of this Viewpoint that ^99m^Tc will lose its central role in imaging due to the lack of contributions from new chemistry. The routine clinical applications of its main players, introduced in general more than 20 years ago, will persist for a time, but nothing new will come. Wherever other d-elements are applied, in catalysis, in materials, in life sciences, or elsewhere, a continuously flowing input from basic studies enables diversity and progress, but little so in ^99^Tc chemistry for the reasons outlined above. Being well aware that funds for basic studies with an element like technetium are sparse, unless something like a “magic bullet” is promised, this author knows that an unexpected finding from chemistry will become an entry. Pure chemistry even with technetium should not abandoned for the sake of application because its beauty, satisfaction, and long-lasting contribution to science are exactly located here.
